# Geriatric Syndromes Across Settings

**DOI:** 10.1093/geroni/igaf122.1321

**Published:** 2025-12-31

## Abstract

Research on geriatric syndromes across settings from the community to acute care setting. Research will be presented on obesity, delirium, dementia and falls.

